# Suicidal behaviour in medicine students and residents

**DOI:** 10.1192/j.eurpsy.2021.1551

**Published:** 2021-08-13

**Authors:** M.D.C. Molina Lietor, I. Cuevas, M. Blanco Prieto

**Affiliations:** Psiquiatría, Hospital Universitario Príncipe de Asturias, Alcalá de Henares, Spain

**Keywords:** Medicine students, Suicide, Depression, suicidal behaviour

## Abstract

**Introduction:**

Among medical students the rate of depression varies between 5-32% and it is known that the suicide rate is higher than in the general population.

**Objectives:**

Clear and current data are needed to design studies for the diagnosis and treatment of students and residents of Medicine with psychological and psychiatric disorders in order to reduce rates of suicidal behavior in this population group.

**Methods:**

A systematic search for articles on the UpToDate, PubMed and Mendeley platforms has been conducted with the keywords “suicide”, “suicidal behavior”, “suicidal ideation”, “medicalschool” and “medical students”. We found 435 items from which a total of 42 items were selected.

**Results:**

The prevalence of depression is 27.2%, of which only 15.7% of them are under treatment. Burnout, has a high prevalence of 45-50%. The prevalence of suicidal ideation in the last year is 11.1%. The most commonly used method is medication overdose. Suicidal behavior is greater among students who choose to choose Psychiatry or Family and Community Medicine as a specialty.

**Conclusions:**

The rate of suicidal ideation among medical students is 11.1%. Nearly one out of every three medical students suffers from depression in some degree, and only one in five is treated. The similar prevalence of burnout at all levels supposes poor management of stress from the faculty. Medical schools should reduce the associated stigma and should encourage depressed students to seek treatment.

